# Maximum Diastolic Potential of Human Induced Pluripotent Stem Cell-Derived Cardiomyocytes Depends Critically on I_Kr_

**DOI:** 10.1371/journal.pone.0040288

**Published:** 2012-07-05

**Authors:** Michael Xavier Doss, José M. Di Diego, Robert J. Goodrow, Yuesheng Wu, Jonathan M. Cordeiro, Vladislav V. Nesterenko, Héctor Barajas-Martínez, Dan Hu, Janire Urrutia, Mayurika Desai, Jacqueline A. Treat, Agapios Sachinidis, Charles Antzelevitch

**Affiliations:** 1 Stem Cell Research and Genomics, Masonic Medical Research Laboratory, Utica, New York, United States of America; 2 Experimental Cardiology, Masonic Medical Research Laboratory, Utica, New York, United States of America; 3 Center of Physiology and Pathophysiology, Institute of Neurophysiology, University of Cologne, Cologne, Germany; University of Milan, Italy

## Abstract

Human induced pluripotent stem cell-derived cardiomyocytes (hiPSC-CM) hold promise for therapeutic applications. To serve these functions, the hiPSC-CM must recapitulate the electrophysiologic properties of native adult cardiomyocytes. This study examines the electrophysiologic characteristics of hiPSC-CM between 11 and 121 days of maturity. Embryoid bodies (EBs) were generated from hiPS cell line reprogrammed with Oct4, Nanog, Lin28 and Sox2. Sharp microelectrodes were used to record action potentials (AP) from spontaneously beating clusters (BC) micro-dissected from the EBs (n = 103; 37°C) and to examine the response to 5 µM E-4031 (n = 21) or BaCl_2_ (n = 22). Patch-clamp techniques were used to record I_Kr_ and I_K1_ from cells enzymatically dissociated from BC (n = 49; 36°C). Spontaneous cycle length (CL) and AP characteristics varied widely among the 103 preparations. E-4031 (5 µM; n = 21) increased Bazett-corrected AP duration from 291.8±81.2 to 426.4±120.2 msec (p<0.001) and generated early afterdepolarizations in 8/21 preparations. In 13/21 BC, E-4031 rapidly depolarized the clusters leading to inexcitability. BaCl_2_, at concentrations that selectively block I_K1_ (50–100 µM), failed to depolarize the majority of clusters (13/22). Patch-clamp experiments revealed very low or negligible I_K1_ in 53% (20/38) of the cells studied, but presence of I_Kr_ in all (11/11). Consistent with the electrophysiological data, RT-PCR and immunohistochemistry studies showed relatively poor mRNA and protein expression of I_K1_ in the majority of cells, but robust expression of I_Kr._ In contrast to recently reported studies, our data point to major deficiencies of hiPSC-CM, with remarkable diversity of electrophysiologic phenotypes as well as pharmacologic responsiveness among beating clusters and cells up to 121 days post-differentiation (dpd). The vast majority have a maximum diastolic potential that depends critically on I_Kr_ due to the absence of I_K1_. Thus, efforts should be directed at producing more specialized and mature hiPSC-CM for future therapeutic applications.

## Introduction

Like embryonic stem cells (ESCs), human induced pluripotent stem cells (hiPSCs) derived by reprogramming somatic cells can be cultivated in the pluripotency state or differentiated into somatic cell types including cardiomyocytes, neuronal cells and insulin producing beta cells of the islets of Langerhans [1], [2]. hiPSC-derived cardiomyocytes (hiPSC-CM) hold promise for use in a variety of applications including: 1) accelerated cost-effective drug development and safety pharmacology; 2) creation of *in vitro* models of genetic diseases to advance our knowledge of pathogenesis as well as to develop patient specific therapeutic modalities [2] (personalized medicine); and 3) regenerative therapy. To serve these functions, the hiPSC-CM must reasonably recapitulate the electrophysiological and pharmacological characteristics of adult native cardiomyocytes.

Based on action potentials (AP) and voltage clamp studies conducted on hiPSC-CM, atrial-like, pacemaker-like and ventricular-like cardiomyocytes have been described and, to a limited extent, ionic currents have been characterized [3]–[5]. However, these studies have been performed with cells isolated in a very early stage of differentiation. In the present study we applied a protocol previously developed for cardiomyogenesis in human ESCs (hESCs) involving stimulation with several growth factors to generate large amounts of cardiomyocyte beating clusters in order to perform a detailed electrophysiological characterization. We analyzed AP characteristics of 103 spontaneously contracting beating clusters (BC) at 11 to 119 days post-differentiation (dpd) and focused on their responses to I_Kr_ and I_K1_ block using E-4031 and BaCl_2_, respectively.

## Results

**Figure 1:**
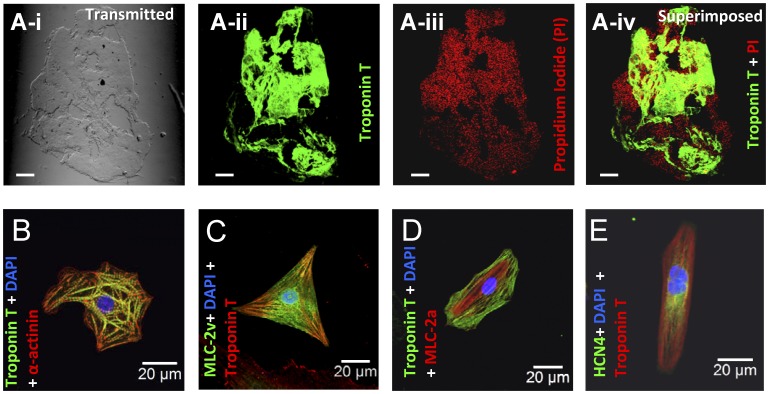
Immuno-labelling of a beating cluster and single hiPSC-CM. Ai-Aiv : Immuno-labelling of a beating cluster, exhibiting contractile activity prior to immunohistochemical processing, with Troponin T specific antibody to visualize cardiomyocytes and propidium iodide to visualize the nuclei of all cells in the BC. The scale bar represents 50 µm, **B-E:** Immuno-labelling of single cells dissociated from a BC with antibodies against canonical pan-cardiac specific marker- Troponin T with α-actinin (B), ventricular myocyte specific MLC-2v (**C**), atrial myocyte specific MLC-2a (D) and pacemaker specific HCN4 (**E**). Scale bars in **B-E** represent 20 µm.

In order to obtain consistent data, we used a directed differentiation protocol to derive cardiomyocytes using serum-free, chemically-defined media supplemented with BMP4, Activin A, bFGF, VEGF and DKK-1 in stage-specific manner as previously described [6], [7]. Our optimized protocol yielded contractile clusters from up to 90% of the total EBs by days 8 to 10 post-differentiation. **Figure 1A** shows the topology of distribution of cardiomyocytes contained in a randomly chosen contractile EB and the enzymatic dissociation of these contractile clusters yielded spontaneously beating single cardiomyocytes as shown in **Figure 1B**. The majority of Troponin T^+^ cardiomyocytes were of a ventricular phenotype (55 to 65%) and the remainder displayed an atrial phenotype based on immunohistochemical (**Figure 1C and D**) and electrophysiological characteristics (described below).

### Characterization of Action Potentials Recorded from Spontaneously Beating Clusters

**Figure 2:**
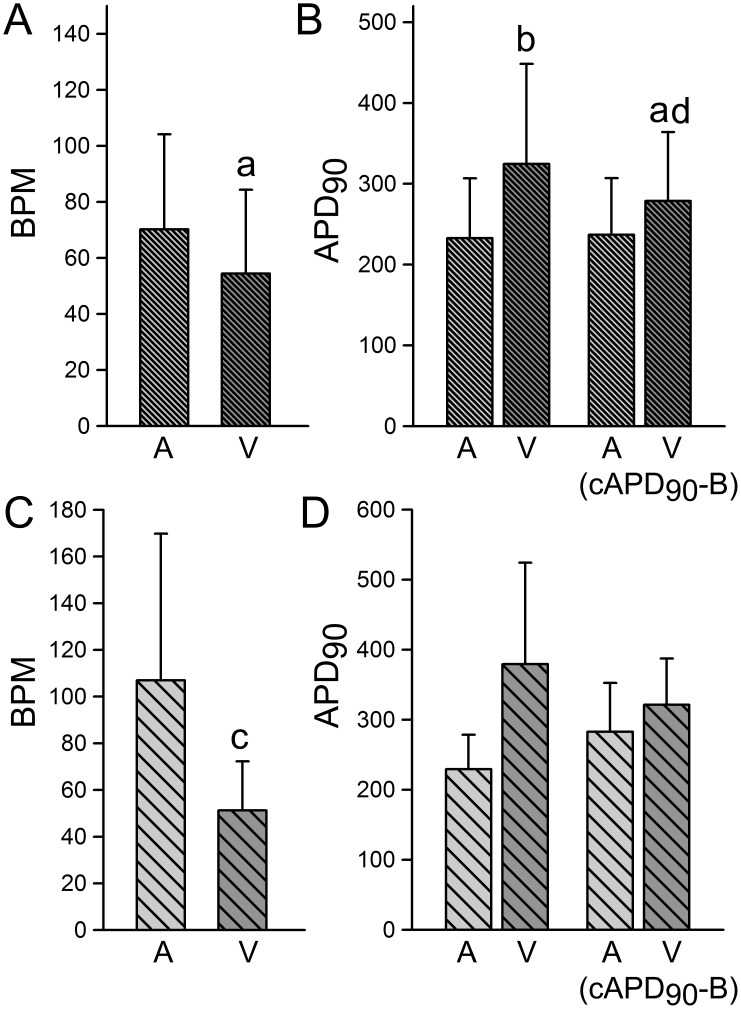
Similarities among beating clusters derived from different embryoid bodies. Similarities between spontaneous rate (BPM; beats/min) and APD_90_ (including cAPD_90_-B) obtained from stable action potentials recordings derived from 87 cardiac beating clusters (BC) (**A** and **B**; 17 batches of EBs) and from 27 BC derived from the same batch of EBs (**C** and **D**). A: Atrial-like; V: Ventricular-like. Atrial- and Ventricular-like action potentials were sorted out on the basis of the APD_30–40_/APD_70–80_ ratios (RO); RO >1.5 = Ventricular. cAPD_90_-B: Bazett’s correction (ADP_90_/(CL)^1/2^). a: p<0.05; c: p<0.001 (vs. Atrial-like).

We obtained stable AP recordings from 103 BC derived from 17 batches of EBs and performed a detailed analysis of their electrophysiological characteristics. In an effort to assess the degree of homogeneity in the electrophysiologic profile of different batches of EBs derived from the same hiPSC line, we compared the spontaneous rate (BPM; beats per min) and action potential duration measured (APD) at 90% repolarization (APD_90_) as well as Bazett’s correction of APD_90_ [cAPD_90_-B]) in hiPSC-CM derived from all 103 BC studied (**Figure 2A and B**; 17 batches of EBs) with those of a single batch in which we studied 27 BC (**Figure 2C and D** **)**. In addition, these data were sorted out by the APD_30–40_/APD_70–80_ ratios (RO) to distinguish between atrial-like (RO≤1.5) and ventricular-like (RO>1.5) APs [8]. The results revealed no significant differences between the two groups suggesting that each batch of EBs-derived BC displays similar electrophysiologic characteristics. However, statistically significant differences were found between all APs parameters when comparing atrial-like vs. ventricular-like cells, except in the maximum diastolic potential (MDP), AP amplitude and V_max_ as shown in **Table 1**. No significant differences were found between all 103 BC (**Table 1A**) and the 27 BC derived from a single batch of EBs (**Table 1B**).

**Table 1:** Summary data of Atrial- and Ventricular-like electrophysiologic parameters.

A	Atrial (n = 40)	Ventricular (n = 63)
Spontaneous Rate (bpm)	70.2±34.0	54.4±30.0^a^
AP amplitude (mV)	100.2±9.8	103.3±12.7
MDP (mV)	−66.2±9.9	−65.9±9.4
V/max (V/sec)	25.7±17.3	24.0±13.8
APD_90_ (msec)	232.9±74.0	324.8±123.7^b^
APD_50_ (msec)	155.1±52.6	263.3±108.5^b^
**Bazett’s-APD_90_** (msec)	237.1±70.1	279.0±85.2^ad^
**Hodges-APD_90_** (msec)	250.7±72.9	315.0±103.3^b^
**Fridericia’s-APD_90_** (msec)	234.3±65.4	290.7±87.4^bd^
**Framingham-APD_90_** (msec)	232.8±74.0	324.7±123.6^b^
APD_50_/APD_90_ (RO)	0.68±0.13	0.81±0.07^b^
APD_30–40_/APD_70–80_ (RO)	1.06±0.36	2.55±0.89^b^
**B**	**Atrial (n = 4)**	**Ventricular (n = 23)**
Spontaneous Rate (bpm)	107.0±62.8	51.3±21.0^c^
AP amplitude (mV)	104.4±7.3	102.7±13.1
MDP (mV)	−65.9±7.3	−64.4±8.7
V/max (V/sec)	32.2±14.9	28.5±17.9
APD_90_ (msec)	229.5±49.0	379.3±145.1
APD_50_ (msec)	150.2±54.2	306.0±127.2^a^
**Bazett’s-APD_90_** (msec)	283.0±69.6	321.5±65.9
**Hodges-APD_90_** (msec)	311.8±84.4	364.1±120.5
**Fridericia’s-APD_90_** (msec)	260.8±39.4	337.4±80.4
**Framingham-APD_90_** (msec)	229.5±48.9	379.2±145.0
APD_50_/APD_90_ (RO)	0.64±0.15	0.8±0.0^b^
APD_30–40_/APD_70–80_ (RO)	1.1±0.5	2.7±0.8^b^

Differences between Atrial-like and Ventricular-like AP parameters derived from 103 BC studied (**A**; 17 batches of EBs) and from 27 BC derived from one batch (**B**). All AP recordings obtained from this later group of 27 BC are depicted in Figure 2. cAPD_90_-H [Hodge’s correction: ADP_90_+1.75 x (heart rate - 60)]; cAPD_90_-Fri (Fridericia’s correction: ADP_90_/(CL)^1/3^); cAPD_90_-Fra [Framingham correction: ADP_90_+0.154 x (1-CL)]. Values are means ± SD. a: p<0.05; b: p<0.001; c: p<0.005 (vs. Atrial-like); d: p<0.05 vs. B.

**Figure 3:**
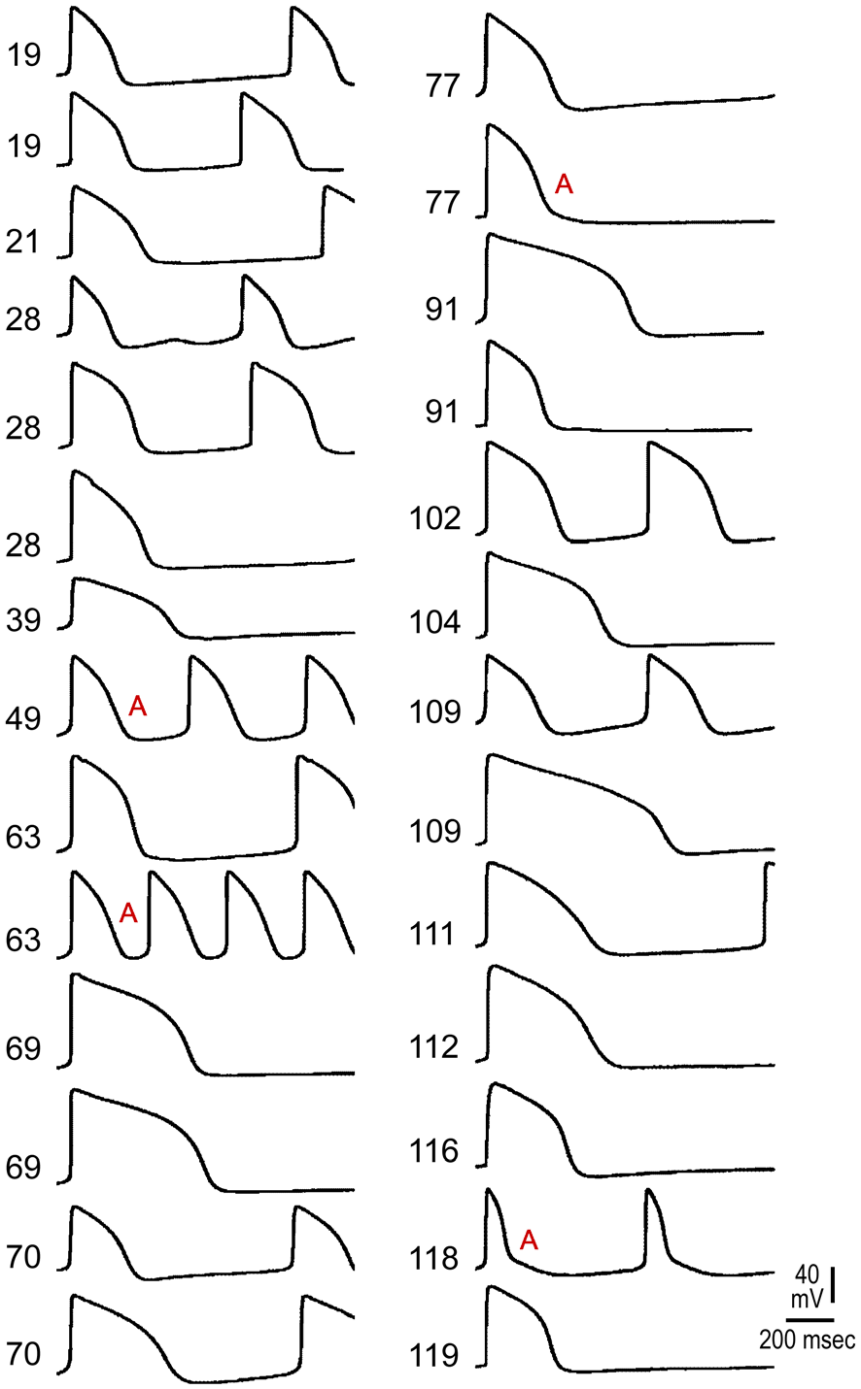
Representative Action Potentials derived from 27 beating clusters obtained from the same batch of embryoid bodies. Action potential (AP) recordings obtained from 27 BC studied from the same batch of EBs. Individual AP parameters are tabulated in **Table 2**. The traces are arranged by the number of days post-differentiation (Age). A: Atrial-like APs (15%); all others were classified as Ventricular-like (85%).

**Figure 3** displays all AP recordings obtained from 27 BC studied from the same batch of EBs. The traces are arranged by the number of dpd (Age; 19 to 119 days). Although 15% (4/27) were classified as atrial-like APs (denoted with an A) and 85% (23/27) as ventricular-like (unmarked APs), the figure reveals a nearly continuous range of AP morphologies, highlighting the subjective nature of distinctions made on the basis of AP profiles. Of note, none of our APs satisfied the criteria for nodal-like (i.e. RO≤1.5+ low amplitude + less negative MDP + low V_max_) [8]. Individual AP parameters (raw data) from these 27 AP recordings, which are sorted out by the APD_30–40_/APD_70–80_ ratios (Atrial-like: RO ≤1.5; Ventricular-like: RO >1.5) are presented in **Table 2**.

**Table 2:** Electrophysiologic parameters from 27 BC of the same batch of EBs.

Atrial-like													
Age(days)	CL(sec)	BPM	Amplitude	MDP	30–40/70–80	APD50	APD90	APD50/APD90	V/max(V/sec)	cAPD90_B	cAPD90_H	cAPD90_Fri	cAPD90_Fra
118	0.7	91.7	103.0	−73.0	0.42	78.0	186.0	0.4	20.0	230.0	241.6	214.3	186.1
77	1.9	32.2	115.0	−71.0	1.10	207.9	298.5	0.7	42.4	218.8	249.9	242.7	298.3
49	0.5	121.0	98.7	−57.7	1.38	166.5	227.2	0.7	18.9	322.7	334.0	287.1	227.3
63	0.3	183.2	101.0	−62.0	1.42	148.4	206.3	0.7	47.7	360.5	421.9	299.3	206.4
**M**	**0.8**	**107.0**	**104.4**	**−65.9**	**1.1**	**150.2**	**229.5**	**0.6**	**32.2**	**283.0**	**311.8**	**260.8**	**229.5**
**SD**	**0.7**	**62.8**	**7.3**	**7.3**	**0.5**	**54.2**	**49.0**	**0.1**	**14.9**	**69.6**	**84.4**	**39.4**	**48.9**

Ventricular-like													
Age(days)	CL(sec)	BPM	Amplitude	MDP	30–40/70–80	APD50	APD90	APD50/APD90	V/max(V/sec)	cAPD90_B	cAPD90_H	cAPD90_Fri	cAPD90_Fra
28	1.0	61.2	84.0	−58.0	1.54	137.0	191.0	0.7	11.0	192.9	193.1	192.3	191.0
109	0.7	89.0	90.0	−58.0	1.87	192.0	258.0	0.7	12.5	314.3	308.8	294.3	258.1
111	1.2	49.8	109.0	−71.0	1.92	343.0	466.0	0.7	32.0	424.5	448.1	437.9	466.0
21	1.1	56.8	89.4	−53.6	1.96	247.8	323.6	0.8	14.1	314.9	318.0	317.7	323.6
70	1.0	60.6	84.9	−47.5	2.06	212.9	277.0	0.8	9.1	278.5	278.2	278.0	277.0
19	1.0	63.2	91.0	−59.0	2.08	163.0	214.0	0.8	15.0	219.6	219.5	217.7	214.0
28	0.8	77.2	105.1	−65.2	2.20	248.2	304.6	0.8	39.8	345.4	334.6	331.3	304.7
102	0.7	87.6	115.0	−70.0	2.21	258.0	318.0	0.8	41.0	384.2	366.3	360.7	318.0
63	0.9	63.7	121.3	−73.5	2.39	243.8	296.8	0.8	69.9	305.9	303.4	302.9	296.8
112	2.0	29.9	118.5	−72.2	2.40	396.3	496.7	0.8	49.5	350.5	444.0	393.7	496.5
77	1.2	51.0	102.6	−70.9	2.43	225.6	291.8	0.8	18.6	269.0	276.0	276.4	291.8
91	1.7	36.3	103.8	−65.0	2.56	218.3	272.2	0.8	18.4	211.7	230.7	230.2	272.1
119	1.3	45.7	100.9	−66.4	2.61	260.0	309.5	0.8	36.7	270.1	284.5	282.6	309.5
70	0.9	66.7	101.0	−53.9	2.69	340.0	434.3	0.8	15.0	457.9	446.0	449.9	434.3
19	0.7	82.8	88.1	−63.1	2.78	202.5	254.7	0.8	39.8	299.1	294.5	283.5	254.7
28	1.2	51.4	112.7	−59.1	2.87	252.0	322.1	0.8	31.5	298.2	307.1	306.0	322.1
116	1.5	39.8	108.0	−71.5	2.92	313.0	371.0	0.8	9.0	302.0	335.6	323.5	370.9
69	1.6	38.7	101.0	−68.0	3.27	312.0	380.0	0.8	17.0	305.2	342.7	328.4	379.9
39	1.6	37.5	75.0	−48.0	3.35	364.0	449.0	0.8	8.0	355.0	409.6	383.9	448.9
104	2.3	26.2	109.1	−59.0	3.49	446.9	531.5	0.8	39.2	351.2	472.3	403.2	531.3
69	2.0	30.8	119.0	−74.0	3.59	441.0	526.0	0.8	40.0	376.7	474.8	421.0	525.9
91	3.0	20.0	119.5	−78.4	4.37	561.7	647.6	0.9	21.8	373.7	577.5	448.9	647.3
109	4.0	14.9	114.0	−74.8	4.84	659.2	788.7	0.8	67.1	392.8	709.7	495.5	788.2
**M**	**1.4**	**51.3**	**102.7**	**−64.4**	**2.7**	**306.0**	**379.3**	**0.8**	**28.5**	**321.5**	**364.1**	**337.4**	**379.2**
**SD**	**0.8**	**21.0**	**13.1**	**8.7**	**0.8**	**127.2**	**145.1**	**0.0**	**17.9**	**65.9**	**120.5**	**80.4**	**145.0**

Control action potential parameters derived from stable recordings obtained from 27 BC of the same batch of EBs. The information is sorted by the APD_30–40_/APD_70–80_ ratio (from the smallest [top] to the largest [bottom]). Age: number of days post-differentiation. The corresponding AP traces are pictured in **Figure 2**. cAPD_90_-B (Bazett’s correction); cAPD_90_-H (Hodge’s correction); cAPD_90_-Fri (Fridericia’s correction); cAPD_90_-Fra (Framingham correction).

**Figure 4:**
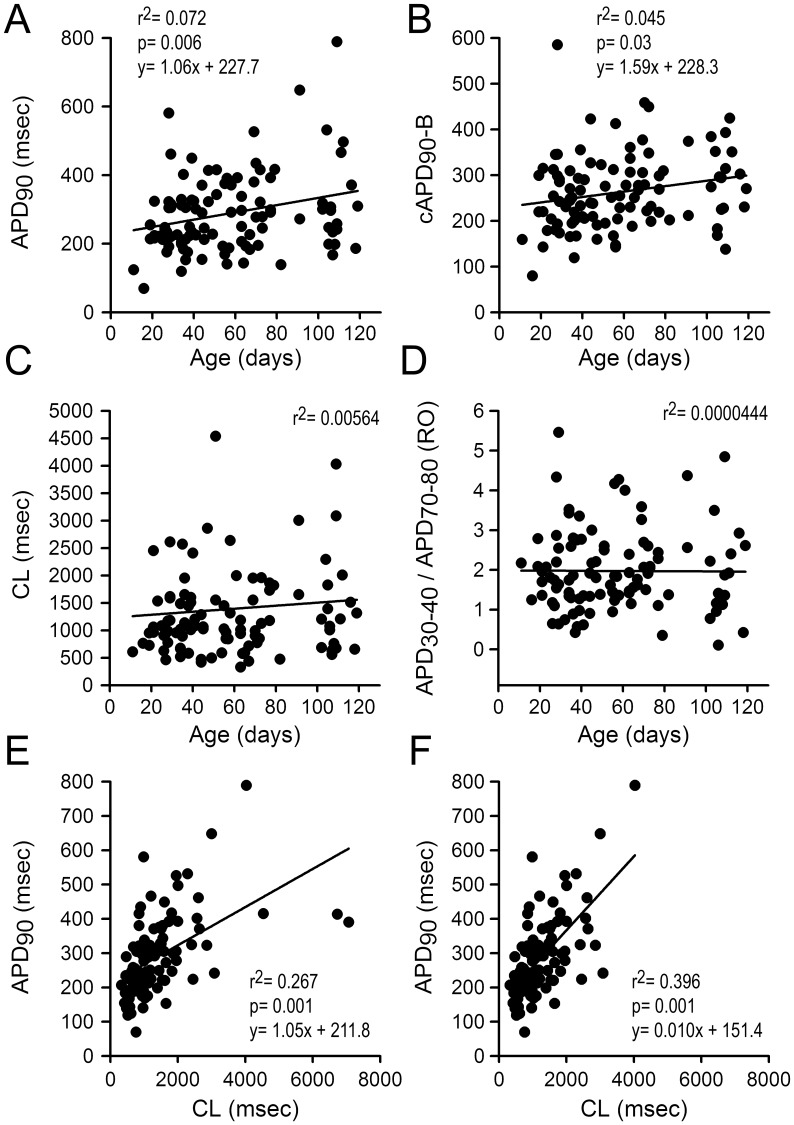
Electrophysiologic parameters as a function of age and cycle length (I). A to D : Action potential parameters as a function of days post-differentiation (Age). **E & F**: APD_90_ as a function of the cycle length (CL). CL range: 327 to 7063 msec; APD_50_ range: 71 to 635 msec; APD_90_ range: 70 to 789 msec; AP amplitude range: 58 to 121 mV; V_max_ range: 5 to 86 V/sec; (n = 103 BC). CL: Cycle Length; APD_50_ and APD_90_: Action potential duration at 50 and 90% repolarization, respectively; AP amplitude: Action potential amplitude; V_max_: dV/dt of Phase 0.

In an effort to expose potential developmental changes, the correlations between APD_90_, cAPD_90_-B, cycle length (CL) and APD_30–40_/APD_70–80_ ratios and dpd (Age) were plotted (**Figure 4A to D** **)**. These results reveal that the APD_90_ as well as the cAPD_90_-B increase as a function of maturity (plots A and B) and that CL as well as the APD_30–40_/APD_70–80_ ratios remain unchanged as a function age (plots C and D). The plots in **Figure 4E and 4F** depict the APD_90_ as a function of the CL with and without outlier’s data, respectively, and show that APD_90_ increases as a function of CL. Each plot in **Figure 4** summarizes electrophysiologic data from the 103 beating clusters studied.

**Figure 5:**
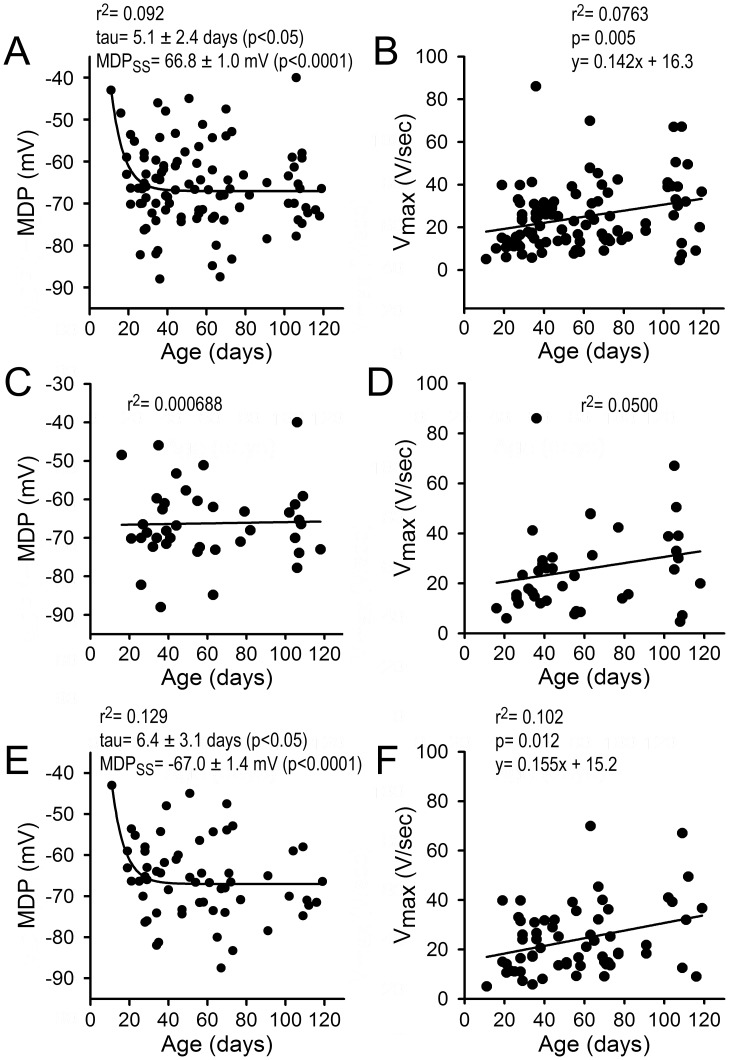
Electrophysiologic parameters as a function of age and cycle length (II). A to D: Action potential parameters as a function of dpd (Age). Relationship between maximum diastolic potential (MDP) or V_max_ and days pos-differentiation (Age) for 103 BC (A and B); 40 BC displaying atrial-like APs (C and D) and 63 BC displaying ventricular-like APs (E and F). The results indicate a significant increase in Vmax as a function of age (panels B and F) as well as a more negative in MDP, particularly in the early post-differentiation period (panels A and E; monoexponential fit).

**Figure 5** plots maximum diastolic potential (MDP) and V_max_ as a function of dpd (Age) for all 103 BC (A and B), for the 40 that display atrial-like APs (C and D) and for the 63 that displayed a ventricular-like profile (E and F). The results reveal an increase in V_max_ as a function of age for all 103 BC (B) and for the 63 displaying a ventricular-like profile (F). The data also reveal a more negative MDP as a function of age (panels A [altogether] and E [ventricular-like]), particularly in the early stages of maturity. No changes in MDP as a function of maturity were found in those BC displaying an atrial-like profile (panel C).

### Effect of E-4031 on Action Potentials Recorded from Spontaneously Beating Clusters

**Figure 6:**
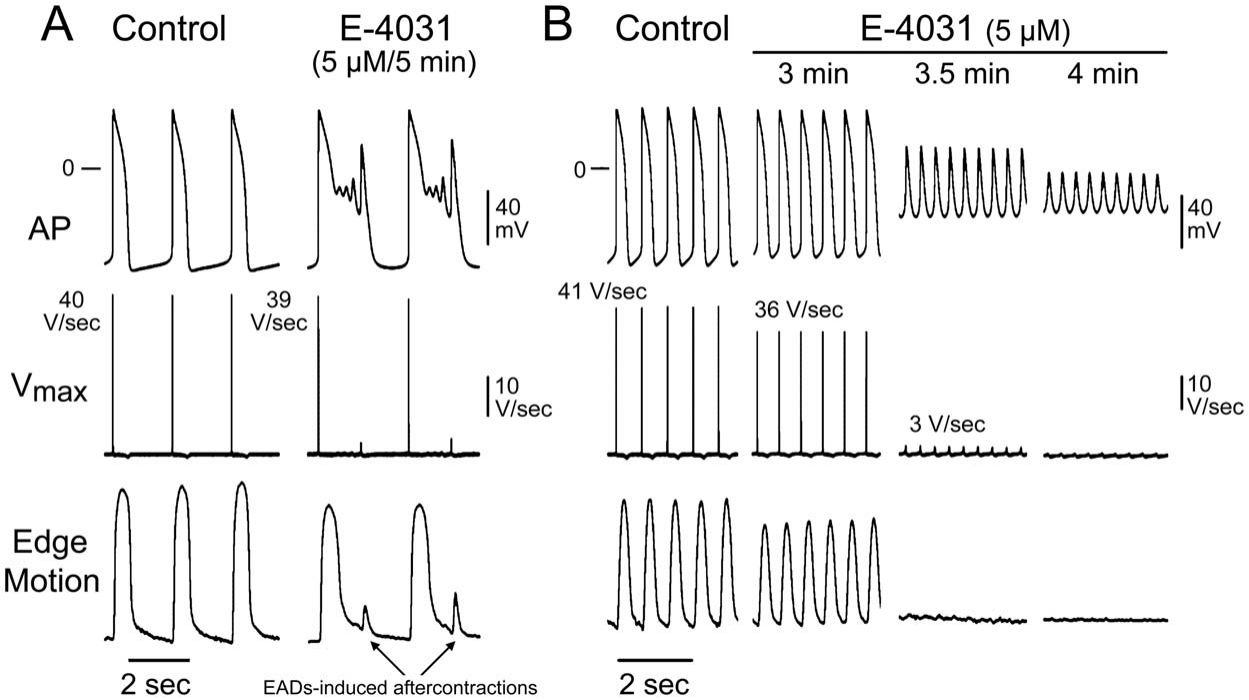
Different electrophysiologic effects of E-4031 in two distinct populations of beating clusters. E-4031-induced I_Kr_ block leads to EADs in cells from some beating clusters, but results in depolarization of cells in the majority of BC. **A and B**: Shown are action potential (AP), V_max_ and contraction (Edge Motion) recordings from a 69 day-old **(A)** and a 102 day-old **(B)** beating clusters under control conditions and following the addition of 5 µM E-4031. In **A**, E-4031 induced EADs within 5 min. In **B**, E-4031 led to depolarization within 3 to 4 min.

**Figure 6** **A** shows AP, V_max_ and contraction recordings from BC at 69 dpd under control conditions and following exposure to 5 µM E-4031 for 5 min. E-4031 led to a dramatic prolongation of APD and development of early afterdepolarizations (EADs). The EADs were accompanied by early aftercontractions. **Figure 6** **B** shows APs, V_max_ and contraction recordings from a BC at 102 dpd under control conditions and following exposure to 5 µM E-4031 for 3–4 min. In this preparation, E4031 depolarized the MDP and increased the frequency of spontaneous activity. **Tables 3A and B** present the electrophysiologic parameters recorded under control conditions and following 5 µM E-4031 from a BC in which this intervention led to EADs (n = 8 [29 to 116 days old]) and from those in which it led to depolarization (n = 13 [25 to 118 days old]). EADs could be readily observed in preparations displaying relatively slow rates and long APDs (**Table 3A**) but not in those presenting with faster rates and shorter APDs (**Table 3B**), which is consistent with the reverse rate-dependence of I_Kr_ block in native cardiomyocytes. Of note, in 3 of 8 preparations, EADs developed just prior to marked depolarization of maximum diastolic potential (data not shown). These observations suggest that MDP is critically dependent on I_Kr_ possibly due to a smaller contribution or lack of I_K1_. Our data also suggest that BC that readily depolarize in response to E-4031 are more deficient in I_K1_ than those that develop EADs.

**Table 3:** Summary data of the effect of E-4031.

A E-4031/EADs (n = 8)		
	Control	E-4031 (5 µM)
Spontaneous Rate (bpm)	32.8±13.9^b^	37.7±10.6^b^
AP amplitude (mV)	107.3±10.5	101.5±11.3^a^
MDP (mV)	−71.0±5.4	−65.9±9.2
V/max (V/sec)	25.0±13.0	21.6±12.8^a^
APD_90_ (msec)	403.4±88.0^b^	692.8±195.1^db^
APD_50_ (msec)	314.9±88.7^e^	366.6±116.0^ab^
**Bazett’s-APD_90_** (msec)	288.3±85.5	530.5±109.1^fb^
**Hodges-APD_90_** (msec)	403.4±88.0^g^	692.8±195.1^db^
**Fridericia’s-APD_90_** (msec)	320.4±81.8	578.4±125.8^fb^
**Framingham-APD_90_** (msec)	403.2±88.0^ g^	692.7±195.1^db^
APD_50_/APD_90_ (RO)	0.8±0.1	0.5±0.1
APD_30–40_/APD_70–80_ (RO)	2.4±1.0	
**B** **E-4031/Depolarization (n = 13)**		
	**Control**	**E-4031 (5 µM)**
Spontaneous Rate (bpm)	65.8±19.4	83.0±39.3^a^
AP amplitude (mV)	103.1±10.4	86.0±21.0^c^
MDP (mV)	−65.3±9.2	−54.2±14.7^d^
V/max (V/sec)	21.3±17.2	15.2±18.2
APD_90_ (msec)	289.9±84.4	337.2±124.3
APD_50_ (msec)	221.9±79.1	209.9±99.3
**Bazett’s-APD_90_** (msec)	294.0±81.9	362.3±73.8^c^
**Hodges-APD_90_** (msec)	289.9±84.4	337.2±124.3
**Fridericia’s-APD_90_** (msec)	291.6±77.9	351.9±87.3^a^
**Framingham-APD_90_** (msec)	289.9±84.3	337.2±124.2
APD_50_/APD_90_ (RO)	0.76±0.12	0.60±0.09
APD_30–40_/APD_70–80_ (RO)	1.9±0.8	

Electrophysiologic parameters measured under control conditions and following 5 µM E-4031 from beating clusters in which this intervention led to EADs (A, n = 8 [29 to 116 days old]) and from those in which it did not (B; n = 13 [25 to 118 days old]). Values are means ± SD. a: p<0.05 vs. Control; b: p<0.001 vs. B; c: p<0.01 vs. Control; d: p<0.005 vs. Control; e: p<0.05 vs. B; f: p<0.001 vs. Control; g: p<0.01 vs. B.

### Effect of BaCl_2_ on Action Potentials Recorded from Spontaneously Beating Clusters

**Figure 7:**
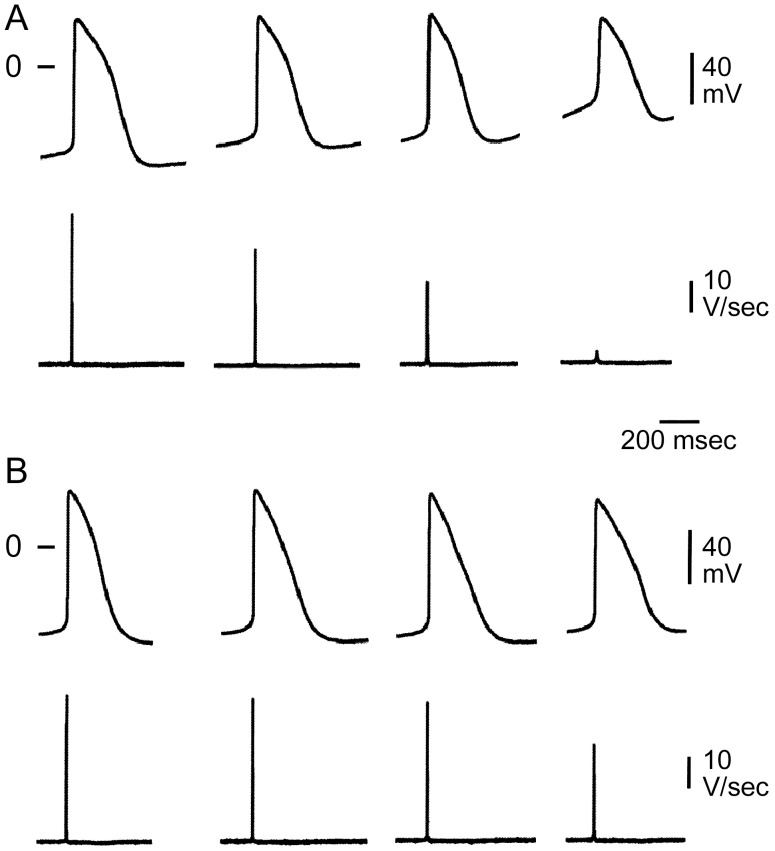
Different e**lectrophysiologic effects of BaCl_2_ in two distinct populations of beating clusters.** Shown are action potentials and V_max_ traces recorded from a 106 day-old (**A**) and a 105 day-old (**B**) beating cluster under control conditions and following the addition of 50, 100 and 500 µM BaCl_2_. In **A**, 50 and 100 µM BaCl_2_ led to membrane depolarization, consistent with the effect of BaCl_2_ to block I_K1_. In **B**, 50 and 100 µM BaCl_2_ induced no changes in MDP, suggesting lack of I_K1_. At a concentration of 500 µM, the APs of both beating clusters depolarized.

As a test of this hypothesis, we exposed BC to BaCl_2_ to inhibit I_K1_. **Figure 7A** shows AP and V_max_ recordings from a 106 day-old BC under control conditions and following 50, 100 and 500 µM BaCl_2_. At a concentration of 50 and 100 µM, BaCl_2_ induced membrane depolarization along with a decrease in AP amplitude and maximum rate of rise of the AP upstroke (V_max_). This effect was consistent with the action of BaCl_2_ to selectively block I_K1_ at a concentration of 100 µM [9]. **Figure 7B** shows AP and V_max_ recordings from a 105 day-old BC in which 50 and 100 µM BaCl_2_ induced little change in MDP, suggesting a markedly reduced level of I_K1_. At concentrations of 500 µM, the APs of both beating clusters depolarized (**Figure 7A and B** **)**. It is noteworthy that at this concentration BaCl_2_ also blocks I_Kr_ [**9**].

**Figure 8:**
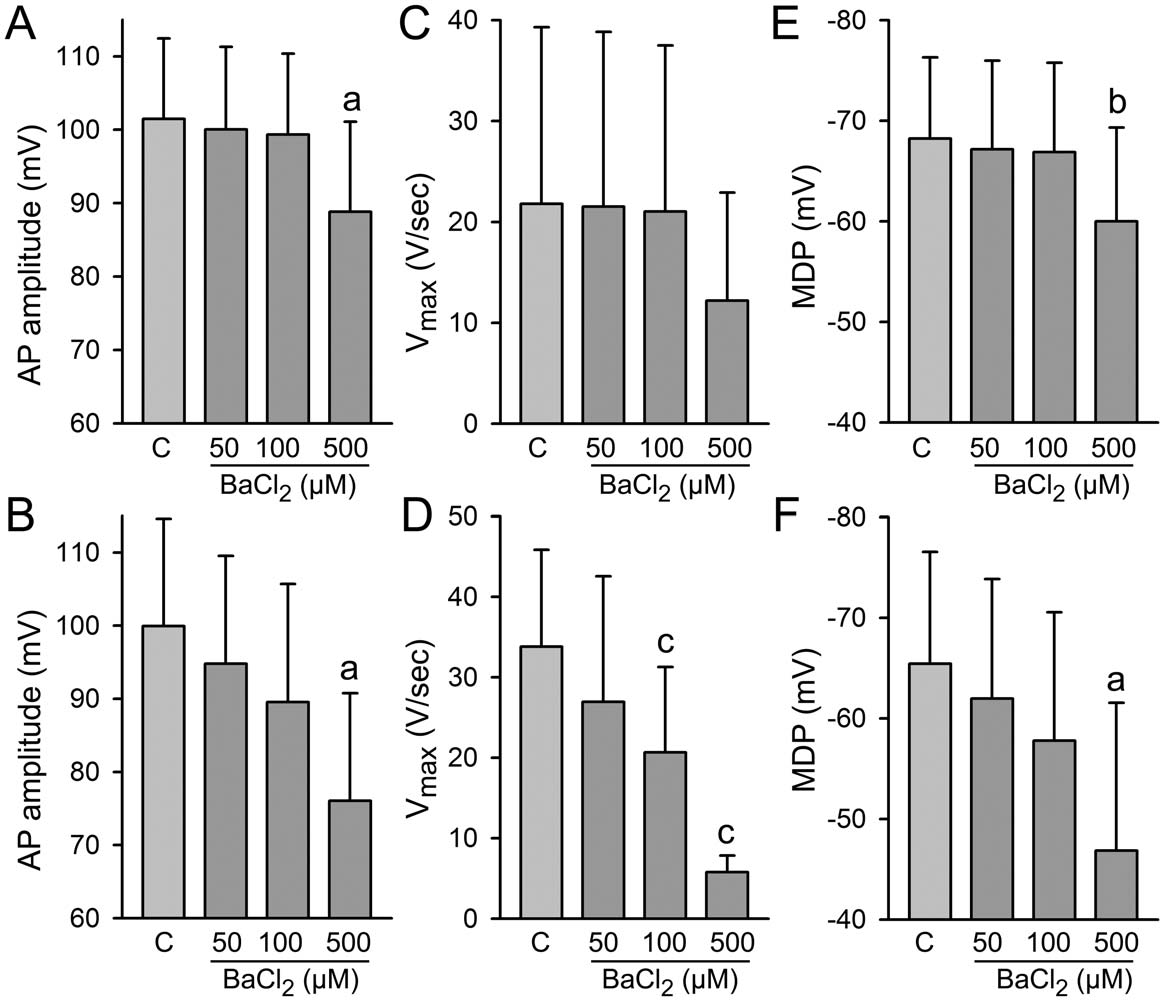
Concentration-dependence of the effect of BaCl_2_ on AP amplitude and V_max_. Concentration-dependence of the effect of BaCl_2_ to reduce AP amplitude, V_max_ and MDP in two population of beating clusters (BC). 100 µM BaCl_2_ induced no changes in MDP in 13 out of 22 BC suggesting a small contribution or lack of I_K1_ (**A,** **C and E**), but led to membrane depolarization in 9 out of 22 BC (**B, D and F**). At concentrations at which BaCl_2_ also blocks I_Kr_ (500 µM), AP amplitude and V_max_ decreased in both groups of BC. a: p<0.05 vs. Control; c: p<0.001 vs. Control.

**Figure 8** shows the concentration-dependence of BaCl_2_ to reduced AP amplitude, V_max_ and MDP in the two populations of BC. In 13 out of 22 BC, 100 µM BaCl_2_ induced little to no change in MDP, suggesting a small contribution or lack of I_K1_ (**Figure 8A, C and E**). In 9 out of 22 BC, 100 µM BaCl_2_ led to membrane depolarization (**Figure 8B, D and F**). At concentrations at which BaCl_2_ also blocks I_Kr_ (500 µM), AP amplitude and V_max_ decreased, and MDP depolarized in both groups of BC.

**Figure 9:**
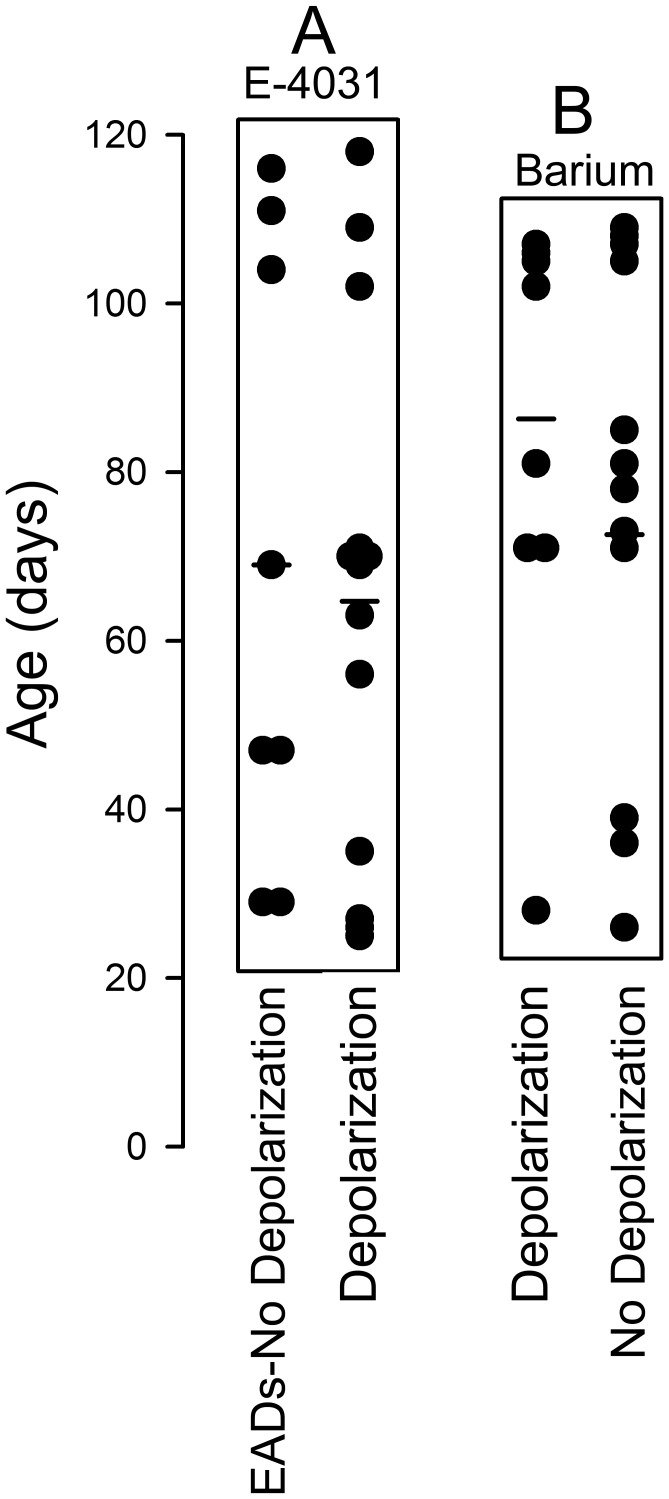
The different electrophysiologic effects of E-4031 and BaCl_2_ is age-independent. A: Range of days post-differentiation (Age) at which E-4031 (5 µM) induced EADs *with little to no change in MDP* vs. those at which it led to depolarization of BC *without exhibiting EADs*. **B:** Range of age of BC that depolarized in response to 100 µM BaCl_2_ (Depolarization) vs. those that did not (No depolarization). Each point represents an individual BC; horizontal lines are the mean values for each group.

The scatter plot illustrated in **Figure 9** shows that these differential electrophysiologic effects of E-4031 and BaCl_2_ are age-independent. In **Figure 9A**, E-4031 (5 µM) led to EADs in BC ranging between 47 and 116 dpd and to depolarization in BC ranging between 56 and 118 dpd. In **Figure 9B**, 100 µM BaCl_2_ led to depolarization in BC ranging between 28 and 81 dpd but not in those ranging between 26 and 85 dpd.

### I_Kr_ Contribution in hiPSC-CM

**Figure 10:**
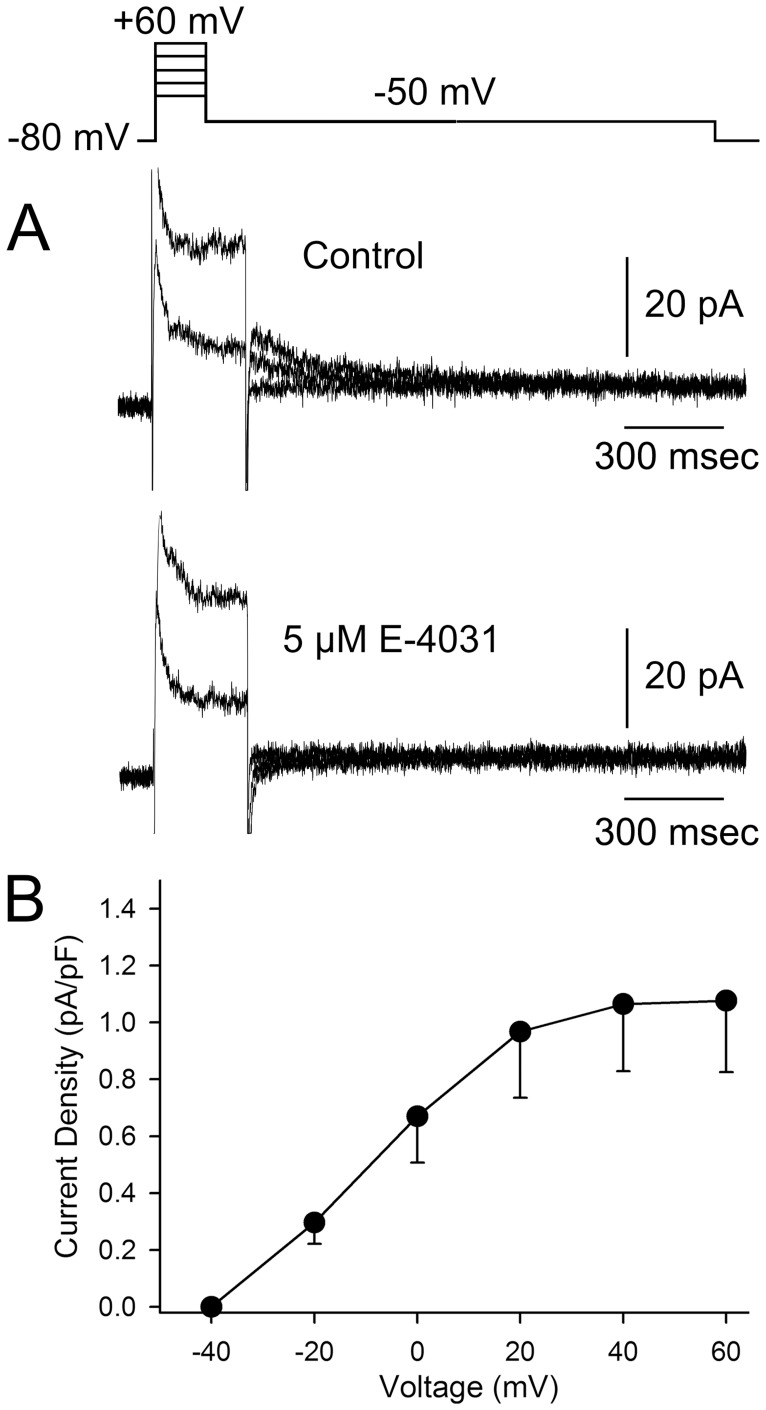
Characteristics of I_Kr_ in hiPSC-CM. A: Representative current traces showing I_Kr_ recorded from hiPSC-derived cardiomyocytes in response to the voltage clamp protocol (top of figure). **B:** Mean I-V relation for I_Kr_ tail current (n = 11).

In native ventricular cells, the rapidly activating delayed rectifier current (I_Kr_) contributes significantly to phase 3 repolarization of the AP. We next measured the magnitude of I_Kr_ in hiPSC-CM (15–114 dpd). Representative traces showing I_Kr_ recorded from hiPSC-CM are depicted in **Figure 10**. I_Kr_ tail currents were measured upon repolarization to −50 mV following application of 300 msec test pulses between −40 to +60 mV in 20 mV increments, as previously described [10] (**Figure 10A**). The amplitude of I_Kr_ tail current reached a plateau at +20 mV and had a density of 1.06±0.24 pA/pF (n = 11, **Figure 10B**). To confirm the identity of the tail currents measured in hiPSC-CM cells, we added the selective inhibitor E-4031 in 4 cells. Application of 5 µM E-4031 abolished the tail currents demonstrating that only I_Kr_ is present under these conditions.

### I_K1_ Contribution in hiPSC-CM

**Figure 11:**
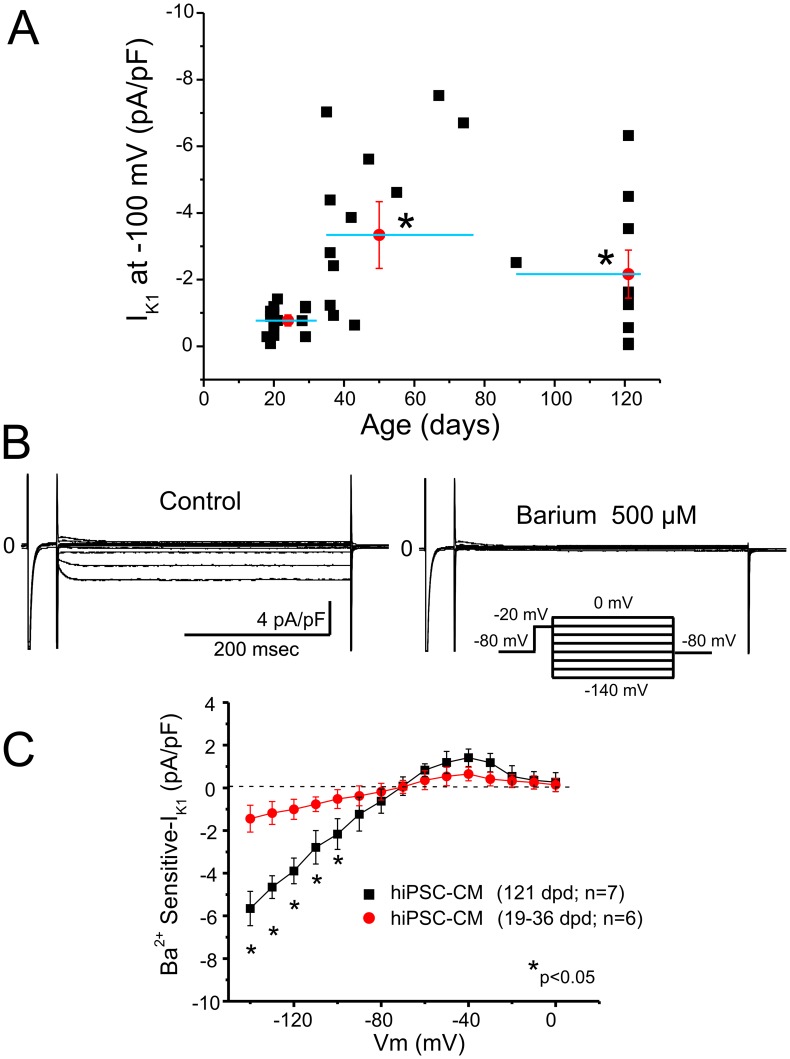
I_K1_ is relatively low or absent in most hiPSC-CM. A : Plot of inward rectifier potassium currents (I_K1_) density in hiPSC-CM obtained at −100 mV hyperpolarizing pulse as a function of age post-differentiation. Red circles denote the mean for early (18 to 29; n = 16 cells), intermediate (35 to 74; n = 12 cells) and late (89 to 121; n = 10 cells) days post-differentiation (dpd), which are delimited by the blue. **B**: Effect of barium: Currents were recorded in hiPSC-CM of 121 dpd under control conditions (left) and in the presence of 500 µM barium (right). Currents were recorded during 400 msec pulses from −140 to 0 mV applied from a holding potential of −80 mV with a prepulse to −20 mV (The voltage protocol is shown in the inset). **C**: I-V relationship of barium-sensitive-I_K1_ obtained by digital subtraction of currents recorded in the absence and presence of BaCl_2_ in hiPSC-CM of 121 and 19–36 dpd. Data were normalized to cell size as reflected by capacitance measurements. Asterisks indicate statistically significant difference between groups (p<0.05).

The contribution of I_K1_ in hiPSC-CM **(** **Figure 11** **)** was evaluated using a standard step voltage clamp protocol. From a holding potential of −80 mV, the cells were depolarized to −20 mV to inactivate I_Na_ and then stepped to membrane voltage between −140 mV and 0 mV for 400 msec in 10 mV increments. A relatively small I_K1_ was observed with a step to −100 mV in isolated cells 18–29 dpd (−0.79±0.097 pA/pF, 16 cells); but a significantly greater I_K1_ was recorded in more mature cells, 35–74 and 89–121 dpd (−3.49±0.91 pA/pF, 12 cells and −2.17±0.72 pA/pF, 10 cells; respectively). **Figure 11A** plots I_K1_ density as a function of age, showing very low levels in the early stage, but significantly larger I_K1_ density at intermediate and late stages of maturity. The effect of barium on I_K1_ (500 µM) was evaluated in hiPSC-CM 121 dpd (**Figure 11B**). Our results indicated that approximately 95% of I_K1_ was barium-sensitive. Over a range of 18 to 121 dpd, I_K1_ density was −2.17±0.42 pA/pF when considering all 38 cells studied with 53% (20 out of 38) showing very low or negligible I_K1_ at −100 mV (<1.8 pA/pF). Although I_K1_ density increases with advancing days post-differentiation, these levels are still considerably less than those observed in native ventricular myocytes [11]. **Figure 11C** shows the I-V relationship of barium-sensitive I_K1_ recorded from hiPSC-CM of 19–36 and 121 dpd. Significant differences were observed between the two age groups.

### Analysis of I_Kr_ and I_K1_ Expression at the mRNA and Protein Levels

**Figure 12:**
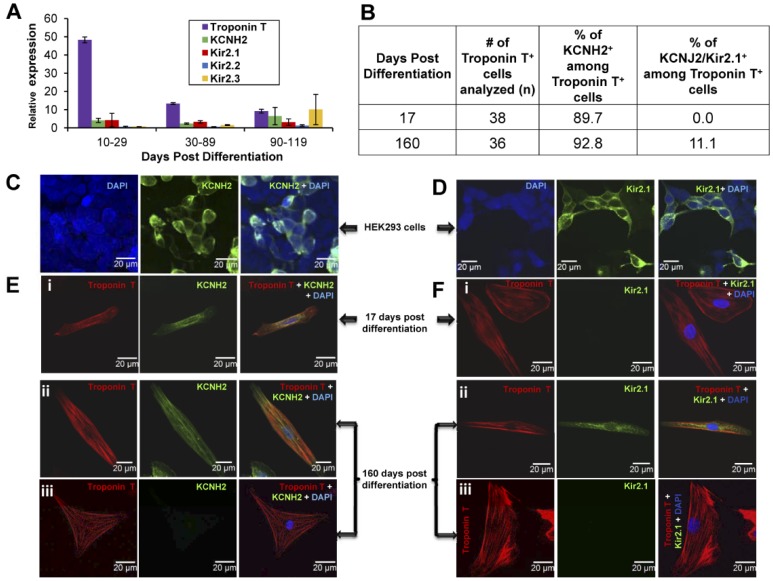
I_Kr_ and I_K1_ expression in hiPSC-CM and beating clusters. A-B: I_Kr_ (KCNH2) and I_K1_ (KCNJ2/Kir2.1) mRNA expression in hiPSC-derived BC at different stages of maturity. **A:** Relative expression levels of KCNJ2/Kir2.1, KCNJ12/Kir2.2, KCNJ4/Kir2.3 and KCNH2 mRNA from the pool of BC. The error bars represent standard error of the mean **B:**Percentage of Troponin T^+^ cardiomyocytes displaying protein expression of KCNH2 (hERG) and Kir2.1 **C-D:** Validation of hERG **(**I_Kr_ **)** and Kir2.1 (I_K1_) antibodies to determine their specificity in HEK293 cells transfected with respective cDNAs. **E-F:** Protein expression of hERG (I_Kr_) and Kir2.1 (I_K1_) in single enzymatically-dissociated cardiomyocytes from BC of 17 and 160 days post-differentiation as analyzed by immunohistochemistry. The majority of Troponin T^+^ hiPSC-CM showed little or no expression of KCNJ2 (I_K1_), whereas over 90% of Troponin T^+^ cells showed hHERG/KCNH2 (I_Kr_) expression at all stages of maturity. Representative Troponin T^+^ cells which stained either positive or negative for either I_Kr_ or I_K1_ in the same immunoslide under identical imaging conditions are shown for cells dissociated from beating clusters 160 dpd. Scale bar represents 20 µm.

Quantitative PCR analysis of total RNA isolated from a pool of beating clusters ranging from 10–119 days post-differentiation revealed expression of KCNJ2/Kir2.1 (the predominant contributor to I_K1_ in the human heart), as well as expression of KCNJ12/Kir2.2, KCNJ4/Kir2.3 and KCNH2 (I_Kr)_ at all stages of maturity, as shown in **Figure 12A**. Because Kir2.x is expressed in cell types other than cardiomyocytes [12] and because BC also contain a diverse array of non-cardiac somatic cells including neuronal and endothelial cells, it is important to recognize that the expression levels of genes encoding Kir2.x may not reflect expression of these genes in cardiomyocytes alone, but in the entire population cells. Indeed, the marked reduction in relative expression of Troponin T suggests that the changes in KCNH2 and Kir2.x message pictured in **Figure 12A** is due largely to expression of these transcripts in other than cardiomyocytes.

We therefore also analyzed the expression of I_Kr_ and I_K1_ in individual cardiomyocytes at the protein level using immunohistochemistry, with Troponin T-specific antibody as a cardiac-specific marker. The enzymatically-dissociated cardiomyocytes were also stained with antibodies against hERG and Kir2.1 to identify I_Kr_ and I_K1_ channels (**Figure 12C and D**). As illustrated in **Figure 12** **,** none of the Troponin T^+^ cells were positive for Kir2.1 (n = 38) at 17 dpd, whereas 11% of Troponin T^+^ cells were positive for Kir2.1 (n = 36) at 160 dpd. The majority of Troponin T^+^ cells exhibited little to no Kir2.1 staining whereas >90% of the Troponin T^+^ cells (n = 74) were positive for hERG at all stages of maturity. Although Kir2.2 and Kir2.3 contribute I_K1_ to some extent in human heart, Kir2.1 is the predominant Kir2.x subunit. In support of this thesis, only Kir2.1 has thus far been identified as a cause of inherited cardiac arrhythmia syndromes associated with a loss of function of I_K1_, such as Andersen-Tawil Syndrome [**13**]. The contribution of Kir2.2 and Kir2.3 has not been studied in great detail in human native cardiomyocytes and requires further experimental explorations.

These data strongly support our electrophysiological findings demonstrating a deficiency of I_K1_ in many hiPSC-CM. It is noteworthy that these Kir2.1-deficient cells display a phenotype similar to that of guinea pig ventricular myocytes that have been transfected with dominant-negative Kir2.1 mutant, which reduces I_K1_ by 50–90% [14].

### Simulated AP Using the Luo-Rudy Phase II (LRII) Model

**Figure 13:**
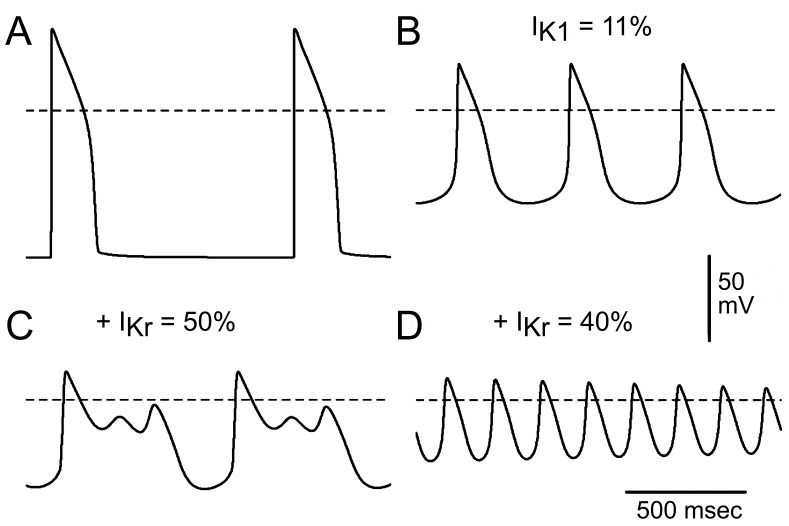
Mathematical model of hiPSC-CM APs. Mathematical model demonstrating that significant reduction of I_K1_ predicts a more depolarized MDP, the appearance of enhanced spontaneous phase 4 depolarization and automaticity as well as a critical dependence of MDP on I_Kr_. **A:** Normal ventricular AP stimulated by the Luo-Rudy II model at a CL of 1000 msec. **B:** When I_K1_ is decreased to 11% of the normal value, AP depolarizes and displays stable automatic activity (MDP is −53.6 mV; CL is 461 msec). **C:** Decreasing I_Kr_ to 50% of the normal value in the presence of 11% I_K1_ results in further depolarization with EADs developing after 20 seconds. **D:** A larger block of I_Kr_ to 40% of the normal value elicits progressively decreasing oscillations of membrane potential leading eventually to the permanent depolarization at −12.8 mV.

We used LRII cellular model to further test the hypothesis that reduced levels or absence of I_K1_ may be responsible for the experimentally observed iPSC-CM automaticity and dramatic effects of I_Kr_ block. **Figure 13A** illustrates the baseline AP produced by the model when stimulated at the CL of 1000 msec. When the maximal conductance of the I_K1_ (G_K1_) is decreased to 11% of its normal value the transmembrane potential promptly depolarizes to −53.7 mV and displays automatic activity as shown in **Figure 13B** (stable automatic APs were obtained 30 sec after G_K1_ decrease in the absence of stimulation; CL = 461 msec). Automaticity develops due to time-dependent deactivation of outward currents (I_Kr_ and I_Ks_) and to voltage-dependent activation of calcium current (I_CaL_), which remains partially activated during depolarized diastolic potentials. In addition, the balance of diastolic currents is affected by outward sodium pump current (I_NaK_) and inward Na-Ca exchange current (I_NaCa_). No automatic activity was produced by the model when G_K1_ was set above 12% of its normal value. On the other hand, a further decrease of G_K1_ to 0% results in additional depolarization (MDP  =  −45.7 mV) and decrease of the CL to 309 msec (not shown). Reduction of I_Kr_ to 50% to mimic blocking effect of E-4031 on this current in the presence of 11% I_K1_ results in further depolarization with EADs developing after 20 seconds, as illustrated on **Figure 13C**. A smaller value of I_Kr_ (40% of the normal value) results in permanent depolarization (MDP  =  −12.8 mV) preceded by potential oscillations around this value as shown in **Figure 13D**. The results of the mathematical model closely recapitulate our experimental observations.

**Table 4:** Summary data of the effect BaCl_2_.

A No Depolarization with 100 µM BaCl2 (n = 13)				
	Control	BaCl2 (50 µM)	BaCl2 (100 µM)	BaCl2 (500 µM)
Spontaneous Rate (bpm)	62.7±27.7	67.7±23.5	68.5±24.9	85.3±23.9
AP amplitude (mV)	101.5±10.9	100.1±11.2	99.3±11.0	88.8±12.2a
MDP (mV)	−68.2±8.1	−67.2±8.8	−66.9±8.9	−60.0±9.3b
V/max (V/sec)	21.8±17.5	21.5±17.3	21.0±16.4	12.2±10.7
APD_90_ (msec)	238.2±57.4	263.2±60.4	266.0±57.3	263.2±50.1
APD_50_ (msec)	176.5±46.9	186.2±47.8	189.6±48.2	178.8±41.0
**Bazett’s-APD_90_** (msec)	231.0±59.1	269.1±50.8	273.4±47.7	305.5±40.8d
**Hodge’s-APD_90_** (msec)	238.2±57.4	263.2±60.4	266.0±57.3	263.2±50.1
**Friederica’s-APD_90_** (msec)	231.9±52.3	266.2±50.0	270.0±46.7	290.1±39.8a
**Framingham-APD_90_** (msec)	238.2±57.4	263.2±60.3	266.0±57.3	263.3±50.1
APD_50_/APD_90_ (RO)	0.74±0.07	0.71±0.09	0.71±0.08	0.68±0.10
APD_30–40_/APD_70–80_ (RO)	1.7±1.4			
**B** **Depolarization with 100 µM BaCl2 (n = 9)**				
	**Control**	**BaCl2 (50 µM)**	**BaCl2 (100 µM)**	**BaCl2 (500 µM)**
Spontaneous Rate (bpm)	74.9±31.9	69.4±20.5	76.2±31.3	73.0±18.3
AP amplitude (mV)	100.0±14.6	94.8±14.8	89.6±16.1	76.1±14.7a
MDP (mV)	−65.4±11.1	−62.0±11.9	−57.8±12.8	−46.9±14.7a
V/max (V/sec)	33.8±12.0	27.0±15.6	20.7±10.6c	5.8±2.1c
APD_90_ (msec)	242.6±64.0	280.0±62.5	276.0±46.3	274.3±42.0
APD_50_ (msec)	152.9±61.9	160.5±48.6	159.8±45.4	160.1±23.3
**Bazett’s-APD_90_** (msec)	257.6±61.8	298.0±73.6	302.9±72.3	297.5±37.9
**Hodge’s-APD_90_** (msec)	242.7±63.9	280.0±62.5	276.1±46.3	274.4±41.9
**Friederica’s-APD_90_** (msec)	251.4±58.3	291.3±66.8	292.3±56.1	289.1±35.1
**Framingham-APD_90_** (msec)	242.6±63.9	280.0±62.5	276.0±46.3	274.3±41.9
APD_50_/APD_90_ (RO)	0.65±0.21	0.60±0.19	0.59±0.15	0.59±0.10
APD_30–40_/APD_70–80_ (RO)	1.1±0.5			

Summarized data of the effect of 50, 100 and 500 µM BaCl_2_ on two populations of BC sorted out by their response to depolarize or not in response to 100 µM BaCl_2_. **A**, BC that did not depolarize (n = 13); **B**, BC that depolarized (n = 9). Values are means ± SD. a p<0.05 vs. Control; b p<0.05 vs. B; c p<0.001 vs. Control; d: p<0.01 vs. Control.

**Table 5:** RT-PCR primer sequences used in this study.

S.No	Gene Name	NCBI Accession ID	Forward Primer	Reverse Primer	Amplicon Size
1	GAPDH	NM_002046.3	CCTGTTCGACAGTCAGCCGCATC	GCGCCCAATACGACCAAATCCGT	111
2	KCNH2	NM_172056.1	GAACGCGGTGCTGAAGGGCT	CCGAAGGCAGCCCTTGGTGG	118
3	KCNJ2	NM_000891.2	CCATGTCCCCATGCTCCTGCG	TCGCACACTGCCCATCGCTT	262
4	Kir2.2	NM_021012.4	GCCTTGAAGACAGTACCGTGCCC	CCGTCCTCCTCCGATGACACGAT	171
5	Kir2.3	NM_152868.1	ACTCTCGTCGGACCCTCCGC	AAGGAAGGCCGCGGAGAAGATCA	266
6	Troponin T	NM_000364.2	GACTGAGCAGACGCCTCCAGGAT	GGGCCATCTTCAGCCTCCTTTGC	266

## Discussion

The ability to utilize hiPSC-CM for safety pharmacology, for the generation of human models of disease or for regenerative therapy requires that these cells reasonably recapitulate the native phenotype. In contrast to recently reported studies, our data point to major deficiencies in this regard, with remarkable diversity of electrophysiologic phenotypes as well as pharmacologic responsiveness among beating clusters and cells derived from human induced pluripotent stem cells.

Multiple impalements from each beating cluster yielded action potentials with similar morphology, suggesting that each cluster was comprised of *one predominant cell type.* In this respect, these results are comparable to those previously described in hESC-derived BC [15]. Because of the small dimension of the BC, it is within the realm of possibility that a diversity of phenotypes is present, but concealed by the electrical coupling characteristics of the functional syncytium [3]. Spontaneous rate and AP characteristics varied widely among the 103 preparations studied indicating a large population heterogeneity: 1) CL range: 327 to 7063 msec; APD_90_ range: 70 to 789 msec; AP amplitude range: 58 to 121 mV; V_max_ range: 5 to 86 V/sec. Using the APD_30–40_/APD_70–80_ RO [8], 39% of the BC displayed atrial-like APs (RO≤1.5) and 61% were ventricular-like (RO >1.5). APD_90_ increased as a function of CL and maturity; V_max_ increased and MDP becomes more negative as a function of age, particularly in the early stages of maturity. A similar large population heterogeneity in the electrophysiologic profile of hiPSC-CM has been described in hESC-derived cardiomyocytes [16].

The ability of E-4031 (5 µM) to induce EADs was, at least in part, related to the intrinsic rate of the beating clusters. EADs could be readily observed in preparations displaying relatively slow rates (mean-rate: 32.8 bpm; mean-CL: 2422.4 msec) and long APDs (mean-APD_90_: 403.4 msec), but not in those presenting with faster rates (mean-rate: 65.8 bpm; mean-CL: 1039.6 msec) and shorter APDs (mean-APD_90_: 289.9 msec). This differential effect is consistent with the reverse rate-dependence actions of I_Kr_ blockers in native cardiomyocytes.

In BC that did not develop EADs (13/21 or 62%), the cells promptly depolarized following 3 to 4 min of exposure to E-4031. These observations suggest that MDP is critically dependent on I_Kr_, possibly due to a smaller contribution or lack of I_K1_.

In support of this hypothesis, BaCl_2_, at concentrations known to selectively block I_K1_ (50–100 µM), failed to depolarize the majority of clusters (13/22 or 59%) and whole cell patch-clamp experiments revealed a very low or negligible I_K1_ in the 53% (20/38) of cells enzymatically dissociated from BC, but the presence of I_Kr_ in all (11/11 or 100%). hiPSC-CM that depolarized in response to I_Kr_ block with E-4031 exhibited a more depolarized MDP and more rapid spontaneous rate **(** **Table 4** **)**. Taken together, these observations suggest that MDP is critically dependent on I_Kr_, due to a small contribution or lack of I_K1_.

Automaticity is a feature common to SA and AV nodal cells but not to native ventricular myocytes. However, myocytes isolated from adult ventricular myocardium have been shown to depolarize and develop automatic activity when exposed to BaCl_2_ (>300 µM) [17], [18]. Consistent with these observations are the results of our mathematical model showing that a reduction in I_K1_ predicts a more depolarized MDP, the appearance of spontaneous phase 4 depolarization and automaticity as well as a critical reliance of MDP on I_Kr_. Moreover, the development of stable EADs without major depolarization in response to I_Kr_ block was only observed in the presence of a relatively robust I_K1_. Thus, the results of the mathematical model closely recapitulate our experimental observations, providing further support for the hypothesis that the absence or deficiency of I_K1_ can account for the immature morphology of the hiPSC-CM APs and their uncharacteristic responsiveness to I_Kr_ blockade.

This study provides a detailed electrophysiologic characterization of hiPSC-CM over the span of over 100 dpd, and supports the conclusion that the majority of the hiPSC-CM do not fully recapitulate the function of adult ventricular cardiomyocytes. In adult cardiomyocytes, regional variations in the density of I_K1_ have been described. I_K1_ is large in ventricular tissue, smaller in Purkinje and atrial tissue and negligible in SA and AV nodal tissue [19]–[21]. Our data suggest that among the most critical electrophysiologic limitations of hiPSC-CM is a deficiency in I_K1_. This deficiency results in marked depolarization when exposed to agents that block I_Kr_. This presents a serious limitation for use of such cells for regenerative therapy because I_Kr_ blockers are ubiquitous in our society. I_Kr_ inhibition is part of the ion channel profile of an ever-growing list and diversity of drugs ranging from diuretics to antidepressants to antiarrhythmics.

The observed deficiency of I_K1_ in hiPSC-CM may be attributable to incomplete developmental or regulation of transcriptional factors mediating *KCNJ2* expression. Additional studies are warranted to ascertain the basis for this deficiency so as to make hiPSC-CM a more reliable *in vitro* model and to harness its full potential for accelerated personalized medicine for a plurality of cardiac diseases.

A potential limitation of our findings is that the deficiency of I_K1_ in our iPSC-CM is protocol-specific. Our protocols are designed to direct cardiac differentiation with a high yield of cardiomyocytes using serum-free media and stage-specific addition of growth factors, similar to protocols used by other investigators worldwide. It is noteworthy that a similar deficiency of I_K1_ has been reported in hESC using other protocols. The data presented in the present study should encourage efforts directed at generating more homogeneous and mature hiPSC-CM phenotypes in which a relatively robust I_K1_ participates in recapitulating native electrophysiologic and pharmacologic behavior. Future studies need to be directed to a molecular understanding of the basis for this deficiency so as to expand the full potential of hiPSC-CM for safety pharmacology, for the generation of human models of disease as well as for advancing the innovative field of cell replacement therapy and heart regeneration.

## Materials and Methods

### Human iPSC Culture and In Vitro Cardiac Differentiation

The human iPS cell line IMR-90-C4 (WiCell, Madison, WI, USA), reprogrammed with Oct4, Sox2, Lin28 and Nanog as described previously, [22] was maintained in serum-free, feeder-free conditions with mTeSR1 media (Stem Cell Technologies, Vancouver, Canada) on BD Matrigel™ (BD Biosciences, CA) coated dishes for routine expansion. We used directed differentiation protocols to derive cardiomyocytes using serum-free, chemically-defined media supplemented with BMP4, Activin A, bFGF, VEGF and DKK-1 in stage specific manner as previously described [6], [7]. Our optimized protocol yielded contractile clusters from up to 90% of the total embryoid bodies by days 8 to 10 post-differentiation. Beating clusters (BC) were micro-dissected from EBs ranging between 11 and 121 days of maturity and plated on gelatin coated dishes with EB10 media (DMEM+GlutaMAX™-I supplemented with 10% Fetal calf Serum pretested for cardiac differentiation (Cat# 100–625, lot# A00C00Z, Gemini Bio-Products, CA), 100 µM MEM Non-essential amino acids and 100 µM β-mercaptoethanol (all except otherwise stated from Gibco, CA). Single cells dissociated with collagenase II (Worthington Biochemical Corp, NY) from the contractile clusters were plated on fibronectin (Invitrogen, CA) coated dishes for single cell electrophysiological recordings.

### Action Potential Recordings

Using sharp microelectrodes (40–60 MΩ when filled with 2.7 M KCl) referenced to ground we characterized stable action potential (AP) recordings at 37±0.5°C from spontaneously beating clusters superfused with HEPES-Tyrode’s solution of the following composition (in mM): NaCl 140, KCl 4, MgCl_2_ 1, HEPES 10, D-Glucose 10 and CaCl_2_ 2; pH was adjusted to 7.4 with NaOH (1N). The microelectrodes were connected to an Axoclamp 2A amplifier (Axon Instruments, Foster City, CA) operating in Bridge mode. In addition, contractility of some beating clusters was assessed using a video edge detection system (model VED 104; Crescent Electronics, Sandy, UT) coupled with a Philips type FTM800NH/HGI camera operating at 60-Hz scan rate. All signals were digitized (sampling rate = 40 kHz), stored on magnetic media and analyzed using Spike 2 for Windows (Cambridge Electronic Design [CED], Cambridge, UK). Following the control recordings, the preparations were exposed to either 5 µM E-4031 (n = 21) or 50, 100 and 500 µM BaCl_2_ (n = 22).

Summary data are reported as mean ± standard deviation (M±SD). Statistical analysis was performed by using *t* test or paired *t* test, or ANOVA as appropriate. A p<0.05 was considered statistically significant.

### Patch-Clamp Recordings

Enzymatically-dissociated hiPSC-CM were superfused with a HEPES buffer of the following composition (mM): NaCl 126, KCl 5.4, MgCl_2_ 1.0, CaCl_2_ 2.0, HEPES 10, and glucose 11. pH was adjusted to 7.4 with NaOH. The patch pipette solution had the following composition (mM): K-aspartate 90, KCl 30, glucose 5.5, MgCl_2_ 1.0, EGTA 5, MgATP 5, HEPES 5, and NaCl 10. pH was adjusted to 7.2 with KOH.

All experiments were performed at 36°C. Cells were placed in a temperature controlled chamber (PDMI-2, Medical Systems Corp.) mounted on the stage of an inverted microscope (Nikon TE300). Voltage clamp recordings were made using a MultiClamp 700A amplifier and MultiClamp Commander (Axon Instruments). Patch pipettes were fabricated from borosilicate glass capillaries (1.5 mm O.D., Fisher Scientific, Pittsburgh, PA). The pipettes were pulled using a gravity puller (Model PP-830, Narashige Corp) and the pipette resistance ranged from 1–4 MΩ when filled with the internal solution. After a whole cell patch was established, cell capacitance was measured by applying −5 mV voltage steps. Electronic compensation of series resistance to 60–70% was applied to minimize voltage errors. All analog signals (cell current and voltage) were acquired at 10–25 kHz, filtered at 2–5 kHz, digitized with a Digidata 1322 converter (Axon Instruments) and stored using pClamp9 software.

Results from pooled data are presented as Mean ± S.E.M. Statistical analysis was performed using an ANOVA test followed by a Student-Newman-Keuls test or a Student t-test, as appropriate. A p<0.05 was considered statistically significant.

### Quantitative Real Time-PCR

qPCR analysis was performed with the ABI Prism 7000 Sequence Detection System (Applied Biosystems, Foster City, CA, USA). Total RNA was extracted with RNAeasy MinElute Cleanup Kit (Qiagen, CA). 100 ng total RNA from each of the pooled clusters ranging from 10–119 days beating clusters were reverse transcribed with SuperScript™ First Strand Synthesis System for RT-PCR (Invitrogen, CA). Real-time PCR was performed in triplicates for every sample using primers listed in **Table 5** using FastStart Universal SYBR Green Master (Rox) (Roche Diagnostics, IN). Averaged C_t_ values of each qPCR reaction from the target gene were normalized with the average C_t_ values of the housekeeping gene *GAPDH*, which ran in the same reaction plate to obtain the ΔC_t_ value. The fold change was calculated as follows: fold change  = **2^–(ΔC^_t1_^−ΔC^_t2_^)^**.

### Immunohistochemistry

Single cells dissociated by trypsin digestion were plated on fibronectin coated dishes and cultured for at least 5 days before immunostaining. The cells were washed with phosphate-buffered saline (PBS) and fixed with 4% paraformaldehyde for 15 minutes. Fixed cells were then permeabilized with 0.1% Triton-X, blocked with 5% fetal calf serum and incubated overnight with primary antibodies followed by 2-hour incubation with the fluorophore-conjugated secondary antibodies in 1∶1000 dilution at room temperature. After the final wash, coverslips were mounted with Prolong Gold Antifade (Molecular Probes, Eugene, OR). Images of labeled cells were collected using Zeiss Laser Scanning Microscope LSM700 and LSM Software Zen2009. The primary antibodies used in this study were anti-Troponin T (Millipore Corp.,1∶300 dilution), α-actinin (Sigma, 1∶200 dilution), MLC-2a (Synaptic Systems, Germany 1;200 dilution), MLC-2v (Synaptic Systems, Germany, 1∶200 dilution), ERG1 (Chemicon, 1∶50 dilution) and Kir2.1 (Chemicon, 1∶50 dilution). The secondary antibodies used were donkey anti-mouse IgG Alexa594, Donkey anti-Mouse IgG Alexa488, Donkey anti-Rabbit IgG Alexa488 (Invitrogen, CA). The Kir2.1 and ERG1 antibodies have been validated for their specificity by staining HEK293 cells transfected with respective cDNA encoding plasmids- pcDNA3.1 KCNJ2 (kind gift from Dr. C. Vandenberg) and pcDNA 3.1 hERG (a kind gift from Dr. A.M. Brown) along with respective isotype control antibodies as shown in Figure 12.C and D. The sub-optimal transfection of HEK293 cells was performed with 0.25 µg plasmid DNA with 3∶1 ratios with Fugene 6 to obtain less than 30% transfection efficiency following manufacturer’s protocol (Roche Diagnostics, IN) to have untransfected cells to serve as negative control in the same immunoslide.

### Computer Simulations

Automatic activity of the iPS derived cardiomyocytes was reproduced using the Luo-Rudy II cellular action potential model [23], [24] by decreasing the maximal conductance of the inward rectifier potassium current (I_K1_) below 11% of normal value. Note that LRII model does not include hyperpolarization-activated inward current (I_f_) and does not exhibit automatic activity under normal conditions. In the absence of the fast upstroke due to inactivation of the fast sodium current (I_Na_) at depolarized diastolic potentials, Ca^2+^ release from the sarcoplasmic reticulum was simulated using “Ca-overload” conditions [25] by fixing G_rel_ at 4 msec^−1^ and timing the start of Ca^2+^ release to the peak of the calcium current (I_CaL_).
